# Supplementary material for: Eating Healthy, Growing Healthy: Outcome Evaluation of the Nutrition Education Program Optimizing the Nutritional Value of Preschool Menus, Poland
Source: Nutrients. 2019 Oct 13;11(10):2438. doi: 10.3390/nu11102438 (PMC6835571; doi:10.3390/nu11102438)
Supplement: Supplementary file 1 [file nutrients-11-02438-s001.pdf]

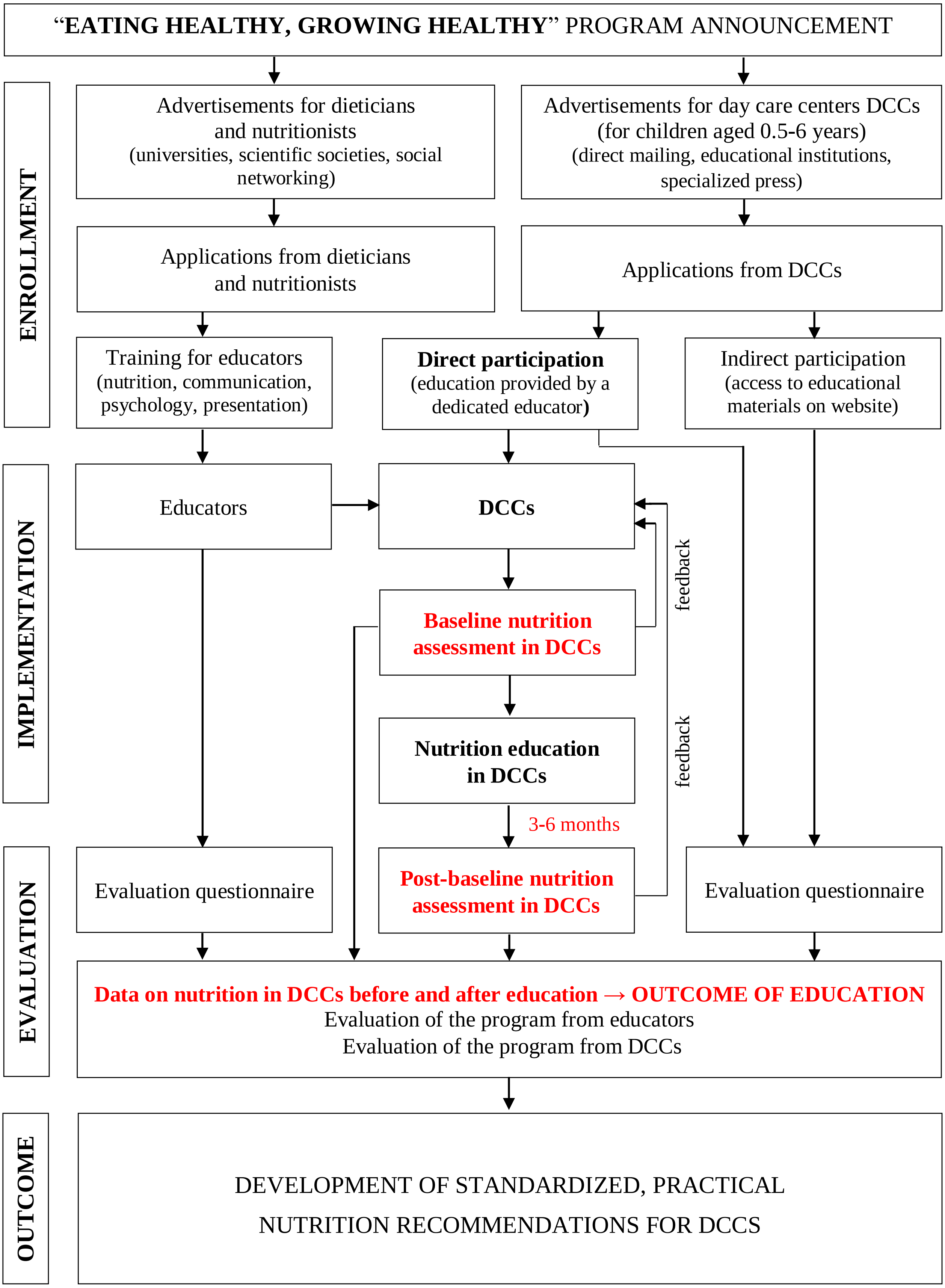

Figure S1. The overall scheme of the program Eating healthy, growing healthy (the part of the program presented in the manuscript is marked in red)

Table S1. The content of energy and nutrients presented as median and 25th and 75th percentiles (per a child a day) in 231 preschools′ 10 day menus before (baseline) and after (post-baseline) the education program.

Nutrient	Daily supply (per a child / day) Median (25th; 75th perc.)	
	**Baseline**	**Post-baseline**
**Energy [kcal]**	1243.1 (1102.2; 1394.2)	1210.9 (1067.7; 1351.5)
**Protein [g]**	44.6 (39.5; 50.6)	44.2 (40.0; 50.6)
**Protein [% of energy]**	14.5 (13.7; 15.5)	15.5 (14.1; 15.7)
**Animal protein [g]**	27.3 (23.9; 31.6)	26.5 (23.8; 30.6)
**Vegetable protein [g]**	17.3 (15.3; 19.8)	14.7 (14.1; 20.2)
**Fat [g]**	44.9 (39.1; 53.0)	43.7 (37.8; 50.2)
**Fat [% of energy]**	33.1 (30.3; 35.5)	33.0 (30.3; 35.1)
**SFA [% of energy]**	12.8 (11.6; 14.1)	12.9 (11.7; 13.9)
**MUFA [% of energy]**	12.9 (11.6; 14.3)	12.7 (11.4; 14.0)
**PUFA [% of energy]**	4.7 (4.1; 5.3)	4.6 (4.1; 5.4)
**Cholesterol [mg]**	165.5 (144.7; 189.8)	162.8 (143.3; 186.2)
**CHO [g]**	161.6 (140.0; 180.5)	157.8 (138.3; 177.4)
**CHO [% of energy]**	51.9 (49.3; 54.5)	51.6 (49.7; 54.1)
**Sucrose [% of energy]**	8.7 (7.0; 11.0)	8.8 (6.5; 10.9)
**Lactose [g]**	10.5 (8.2; 13.0)	10.8 (8.6; 13.0)
**Starch [g]**	93.0 (81.9; 106.1)	92.5 (80.1; 107.0)
**Dietary fiber [g]**	15.5 (13.6; 18.3)	16.0 (13.6; 18.3)
**Vitamin A [µg]**	1019.7 (750.0; 1285.5)	1023.9 (815.1; 1310.2)
**Retinol [µg]**	256.3 (212.7; 312.7)	249.8 (207.6; 310.5)
**Beta-carotene [µg]**	4074.2 (3047.8; 5508.8)	4340.3 (3280.9; 5855.9)
**Vitamin B_1 [mg]**	0.9 (0.8; 1.0)	0.9 (0.8; 1.0)
**Vitamin B_2 [mg]**	1.1 (0.9; 1.2)	1.1 (1.0; 1.2)
**Vitamin B_6 [mg]**	1.5 (1.3; 1.7)	1.5 (1.3; 1.7)
**Vitamin B_{12} [µg]**	2.6 (2.2; 3.2)	2.6 (2.1; 3.1)
**Vitamin C [mg]**	98.4 (81.1; 117.4)	99.5 (83.2; 122.7)
**Vitamin D [µg]**	1.7 (1.3; 2.1)	1.6 (1.2; 2.0)
**Vitamin E [mg]**	6.3 (5.2; 7.5)	6.3 (5.2; 7.3)
**Folate [µg]**	214.1 (184.8; 249.1)	218.0 (185.5; 253.9)
**Niacin [mg]**	10.2 (8.9; 11.3)	10.0 (8.7; 11.5)
**Calcium [mg]**	450.7 (373.5; 528.5)	457.9 (388.8; 535.0)
**Copper [mg]**	0.8 (0.7; 0.9)	0.8 (0.7; 1.0)
**Iodine [µg]**	97.3 (69.2; 135.7)	96.8 (66.4; 133.7)
**Iron [mg]**	6.5 (5.7; 7.4)	6.5 (5.8; 7.6)
**Magnesium [mg]**	200.6 (175.9; 230.4)	202.8 (179.3; 232.0)
**Phosphorus [mg]**	792.4 (705.6; 887.5)	786.5 (712.4; 900.8)
**Potassium [mg]**	2439.3 (2119.1; 2769.2)	2381.9 (2107.1; 2764.2)
**Sodium [mg]**	2046.6 (1567.0; 2720.4)	2046.0 (1439.5; 2632.0)
**Zinc [mg]**	5.7 (5.1; 6.6)	5.8 (5.3; 6.7)
